# The Perspectives of General Nephrologists Toward Transitions of Care and Management of Failing Kidney Transplants

**DOI:** 10.3389/ti.2023.11172

**Published:** 2023-06-30

**Authors:** Tarek Alhamad, Haris Murad, Darshana M. Dadhania, Martha Pavlakis, Sandesh Parajuli, Beatrice P. Concepcion, Neeraj Singh, Naoka Murakami, Michael J. Casey, Mengmeng Ji, Michelle Lubetzky, Ekamol Tantisattamo, Omar Alomar, Arman Faravardeh, Christopher D. Blosser, Arpita Basu, Gaurav Gupta, Joel T. Adler, Deborah Adey, Kenneth J. Woodside, Song C. Ong, Ronald F. Parsons, Krista L. Lentine

**Affiliations:** ^1^ John T. Milliken Department of Medicine, Washington University in St. Louis, Saint Louis, MO, United States; ^2^ Department of Transplantation Medicine, Weill Cornel Medicine - New York Presbyterian Hospital, New York, NY, United States; ^3^ Department of Medicine, Beth Israel Deaconess Medical Center and Harvard University, Boston, MA, United States; ^4^ Department of Medicine, University of Wisconsin - Madison, Madison, WI, United States; ^5^ Department of Medicine, University of Chicago, Chicago, IL, United States; ^6^ John C. McDonald Regional Transplant Center, Willis Knighton Health System, Shreveport, LA, United States; ^7^ Department of Medicine, Brigham and Women’s Hospital and Harvard Medical School, Boston, MA, United States; ^8^ Department of Medicine, Medical University of South Carolina, Charleston, SC, United States; ^9^ Division of Abdominal Transplantation, Department of Surgery and Perioperative Care, Dell Medical School, University of Texas at Austin, Austin, TX, United States; ^10^ Department of Medicine, University of California, Irvine, Orange, CA, United States; ^11^ SHARP Kidney and Pancreas Transplant Center, San Diego, CA, United States; ^12^ Department of Medicine, Seattle Children’s Hospital, University of Washington, Seattle, WA, United States; ^13^ Department of Medicine, Emory University, Atlanta, GA, United States; ^14^ Department of Medicine, Virginia Commonwealth University, Richmond, VA, United States; ^15^ Department of Medicine, University of California, San Francisco, San Francisco, CA, United States; ^16^ Department of Surgery, University of Michigan, Ann Arbor, MI, United States; ^17^ Department of Medicine, University of Alabama at Birmingham, Birmingham, AL, United States; ^18^ Center for Abdominal Transplantation, Saint Louis University, Saint Louis, MO, United States

**Keywords:** re-transplantation, failing kidney allograft, transition of care, immunosuppression management, multidisciplinary team

## Abstract

The management of failing kidney allograft and transition of care to general nephrologists (GN) remain a complex process. The Kidney Pancreas Community of Practice (KPCOP) Failing Allograft Workgroup designed and distributed a survey to GN between May and September 2021. Participants were invited via mail and email invitations. There were 103 respondents with primarily adult nephrology practices, of whom 41% had an academic affiliation. More than 60% reported listing for a second kidney as the most important concern in caring for patients with a failing allograft, followed by immunosuppression management (46%) and risk of mortality (38%), while resistant anemia was considered less of a concern. For the initial approach to immunosuppression reduction, 60% stop antimetabolites first, and 26% defer to the transplant nephrologist. Communicating with transplant centers about immunosuppression cessation was reported to occur always by 60%, and sometimes by 29%, while 12% reported making the decision independently. Nephrologists with academic appointments communicate with transplant providers more than private nephrologists (74% vs. 49%, *p* = 0.015). There are heterogeneous approaches to the care of patients with a failing allograft. Efforts to strengthen transitions of care and to develop practical practice guidelines are needed to improve the outcomes of this vulnerable population.

## Graphical Abstract

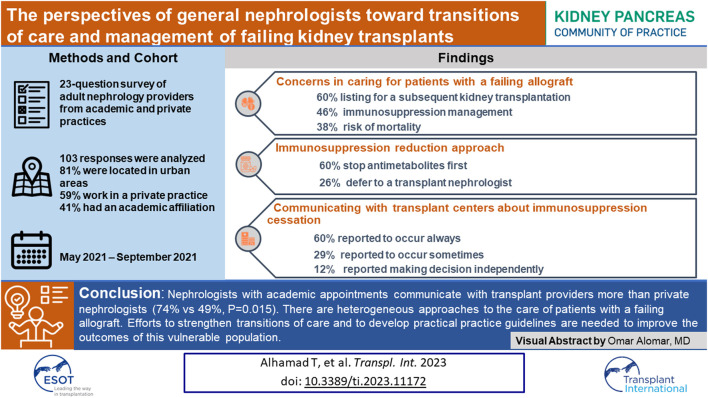

## Introduction

Kidney transplants have a limited life span, with a median half-life ranging from 9 to 12 years [1, 2]. In fact, 11.9% of patients on the kidney transplant waiting list have had a prior failed transplant [3]. These patients are at a higher risk of morbidity and mortality compared to patients who are on dialysis without a previous failed transplant [4–6]. This increased risk is thought to be due to a combination of immunocompromised status, as well as a chronic inflammatory state which leads to increased infectious and cardiovascular complications, amongst others [7–9].

Effective transitions of care between providers are an ongoing challenge in chronic kidney disease (CKD) [10]. Timely referral of patients with failing allografts to general nephrologists is crucial to begin appropriate CKD care. Based on the care model, CKD care may not be the focus of some transplant centers. This includes vascular access planning, anemia management, and a well-timed transition to dialysis. Patients with a failing allograft face many challenges, including high risk of depression and social challenges that would add more difficulties in their care.

The American Society of Transplantation Kidney Pancreas Community of Practice (AST-KPCOP) established a workgroup to study Kidney Recipients with Allograft Failure–Transition of Care (KRAFT) to understand the current data and practice patterns related to the management of recipients with a failing allograft. A recent survey of transplant providers performed by this group reflected the common transition-related practices of transplant providers and highlighted the substantial heterogeneity in several aspects of the care of patients with allograft failure [9]. To date, the practice patterns of general nephrology providers related to this important area of kidney patient care has not been studied. Hence, we conducted a survey to assess practice patterns and priorities of general nephrology providers regarding patients with a failing or failed kidney allograft.

## Methods

### Survey Design

This survey was performed by AST-KPCOP’s KRAFT workgroup. The survey questions were developed collaboratively and the instrument was piloted with general nephrologists as well as among KPCOP workgroup members. Where needed, the wording of the questions was adjusted for clarity. The final survey comprised 23 questions, including two related to management of failing allografts, six related to the comfort of and approach to tapering immunosuppression, three related to perceived risks and benefits associated with tapering immunosuppression, four related to communications and referrals to transplant centers, three related to the management of rejection in a failed allograft, and five related to program description which included practice type, size, and location. Main concerns in the care of patients with a failing kidney allograft and factors linked to tapering off immunosuppression were graded on a semi-quantitative scale: very important, intermediate importance, and not very important.

### Survey Administration and Participants

The survey was approved by the Washington University in St. Louis Institutional Review Board and approved by the Education Committee of AST and KPCOP for distribution. The survey was built into the SurveyMonkey tool and distributed in May 2021 via mail to the members (MD, NP, AP) of the American Society of Nephrology (ASN), electronic links by individual email invitations to general nephrologists in academic and private institutions, and by posting the link in the KPCOP HUB. The survey pool was closed on 1 September 2021. Responses were recorded anonymously. Zip codes were used to examine the distribution of responses in the US.

ArcMap 10.8 was used to geocode the location of respondents using 5-digit zip code locator. The Zip codes of the respondents were then linked to a reference map of 2020 Census Bureau’s urban area classification to identify the rural and urban areas. Chi-square test was performed for comparison of responses across practice type and geographical area.

## Results

### Survey Participants

There were 66 responses who received the invitations from the mailing cards to the ASN members and 56 responses from electronic links from individual email invitations and through the link in the KPCOP HUB. In total, we registered a total of 122 responses. We excluded two responses from providers that were primarily pediatrics and 17 responses from providers whose practice included more than 50%, transplant patients. Amongst the remaining 103 responses, 98 responses were from the US, one from Canada, and four from other countries (2 Pakistan, 1 Belgium, and 1 Singapore).

Of the 103 responses who practiced primarily adult nephrology, 41% had an academic affiliation. 23% practiced in a small private practice with one to four nephrologists, 16% practiced in a medium-size practice with five to ten nephrologists, and 20% practiced in a large private practice with more than 10 nephrologists. Most participating nephrologists were located within urbanized areas.

### Patients With a Failing or Failed Kidney Allograft

More than half (*n* = 57) reported that only 1%–5% of dialysis patients in their practice had failed kidney allografts, one third reported 6%–10%, eight reported 11%–20%, one reported more than 20%, and only three reported that their practice did not include any failed or failing kidney allografts.

### Communication and Transition of Care With a Failing Allograft

**FIGURE 1:**
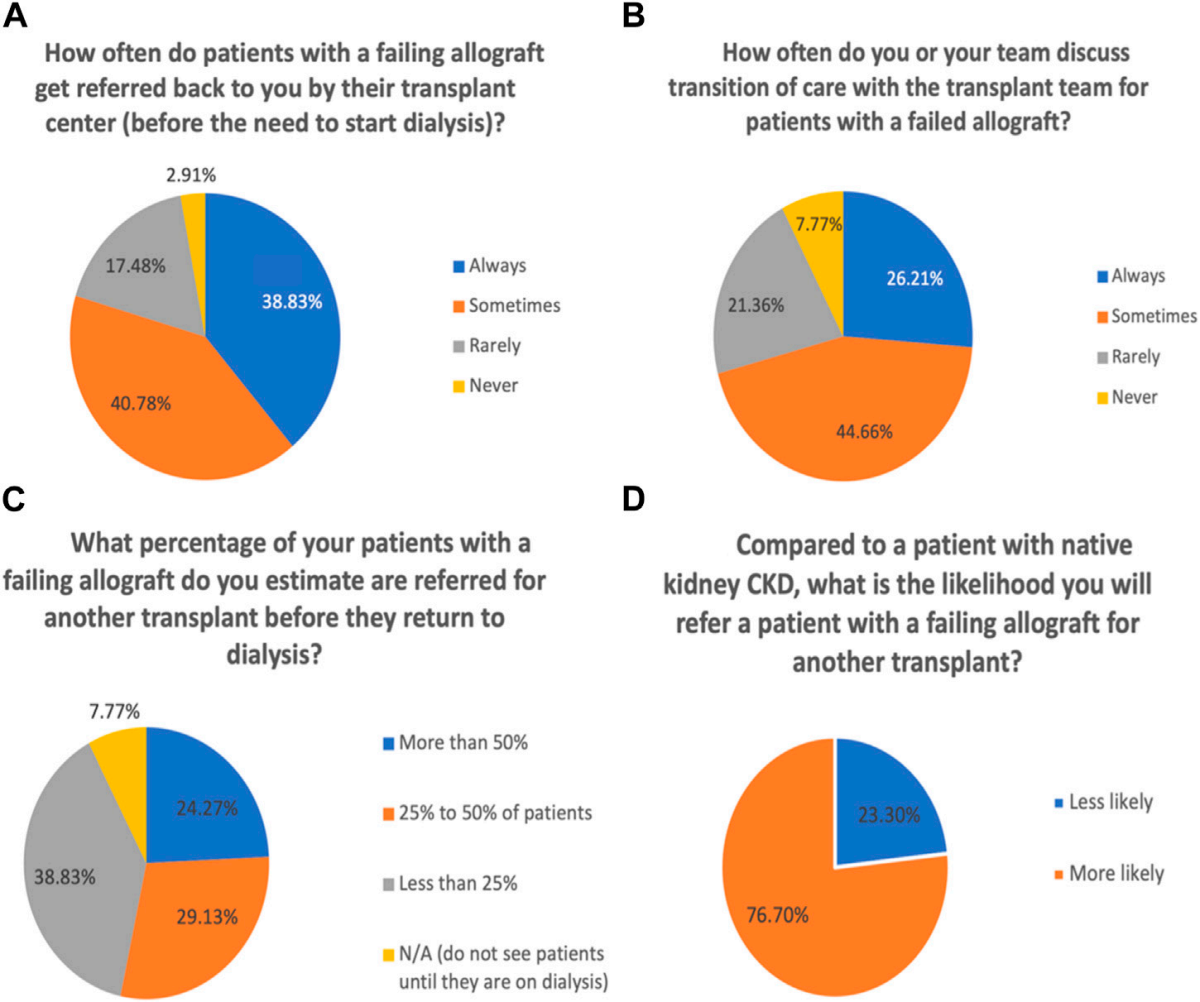
Communications with transplant nephrology and referral patterns for patients with failing allografts. **(A)** Referrals back to general nephrologists before starting dialysis. **(B)** Discussion of transition of care with transplant teams. **(C)** Rate of referrals for another transplant before return to dialysis. **(D)** Referrals of patients with failing allograft to transplant compared to patients with native CKD.

Please see Figure 1 for all responses and results. When asked how often patients with a failing allograft get referred back to general nephrologists by their transplant center, 39% reported always (Figure 1A). Regarding discussing the transition of care with the transplant team for patients with a failed allograft, 26% of respondents reported always (Figure 1B).

In terms of referral for another transplant before return to dialysis, 24% of respondents reported that more than 50% of the patients with a failing allograft were referred (Figure 1C). The majority (77%) of respondents were more likely to refer a patient with a failing allograft than a patient with native CKD for another transplant (Figure 1D).

### Immunosuppression Approach With a Failing Allograft

Respondents were asked if they feel comfortable managing immunosuppression for patients with a failing allograft (not on dialysis). 22% felt very comfortable, 60% felt comfortable unless complications developed, and 18% were uncomfortable and would need guidance.

In terms of the initial approach for reduction of immunosuppression in a patient with a failing allograft, a majority, 60% of respondents would stop antimetabolites first, 26% would defer to transplant nephrologist, 8% would stop calcineurin inhibitors first, and 6% would stop prednisone first.

### Reasons and Concerns for Maintaining Immunosuppression

Approximately 30% of respondents believed that continuing immunosuppression in a patient who has started dialysis would increase the risk of having adverse events and/or mortality, 35% did not think continuing immunosuppression increases risks, and the remaining 35% were not sure. None of the respondents would stop all immunosuppression when a patient with a failing allograft starts dialysis, 55% would taper off immunosuppression, while 46% would continue immunosuppression.

**FIGURE 2:**
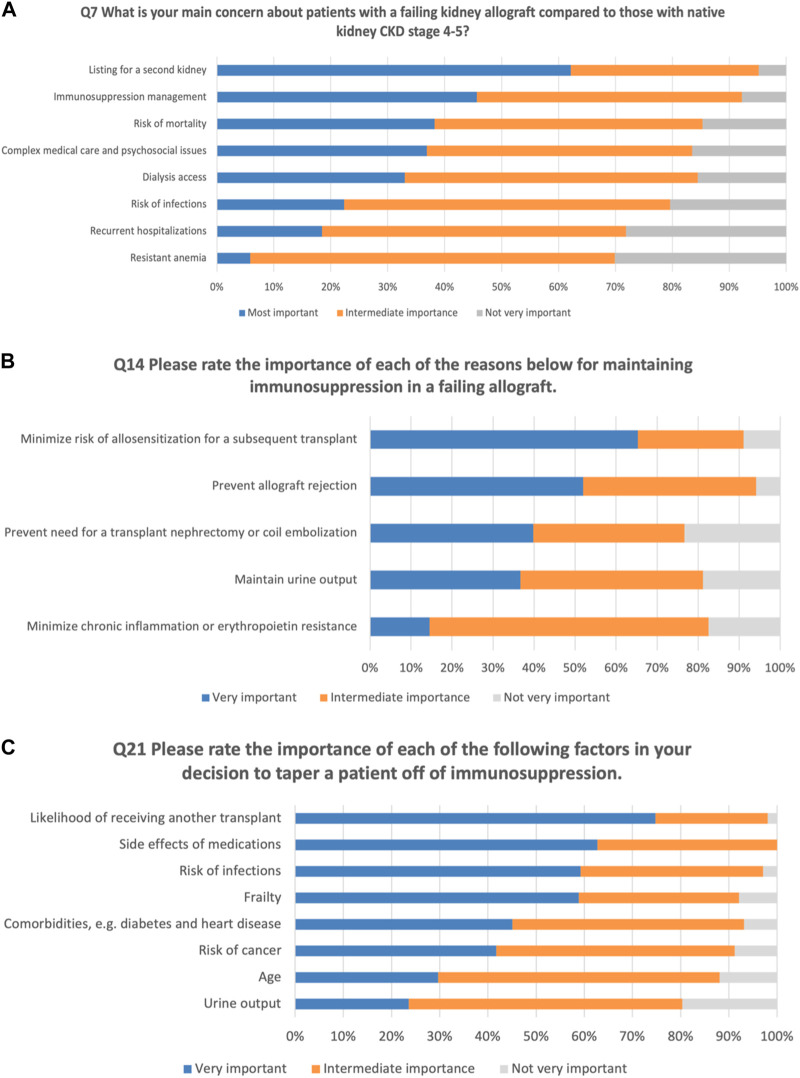
Perception of general nephrologists on the management of patients with failing kidney allograft. **(A)** Concerns about patients with failing allografts compared to native CKD. **(B)** Importance of selected factors in maintaining immunosuppression. **(C)** Importance of selected factors in the decision to taper immunosuppression.

Compared to care for those with native kidney CKD stage 4–5, more than 60% of respondents reported that listing for a second kidney is the most important concern for patients with a failing allograft, followed by immunosuppression management (46%). Other responses could be seen in Figure 2A.

The respondents were asked to rate the importance of reasons for maintaining immunosuppression in a failing allograft. About 65% reported that minimizing the risk of allosensitization for a subsequent transplant is the most important factor in their decision to maintain immunosuppression, followed by preventing allograft rejection (52%) (Figure 2B).

### Immunosuppression Reduction When Starting Dialysis

Respondents were asked if they communicate with transplant centers regarding immunosuppression cessation in patients with a failing allograft. About 60% reported always communicating, 29% reported sometimes, and 12% reported that they made the decision themselves. Among 93 respondents who manage immunosuppression, 73% monitor calcineurin inhibitor levels, and 72% monitor urine output in deciding when to stop immunosuppression.

The respondents were asked if their approach towards immunosuppression in a failed allograft would be different when the patient’s waiting time for a kidney is less than 1 year (e.g., availability of a living donor) compared to longer waiting times. 48% of respondents indicated they would keep patients at a more intensive regimen if the patient’s waiting time for a kidney is <1 year; 10% said that the presence of a living donor would not alter their immunosuppression plans; 43% said that they would defer to transplant nephrology.

We examined the essential factors that influenced clinicians’ decisions for tapering off immunosuppression. More than 75% reported that the likelihood of receiving another transplant is the most important factor in their decision to taper off immunosuppression, followed by side effects of medications (63%). The least important factors were reported to be urine output and the age of the patient (Figure 2C).

### Referral for Allograft Nephrectomy

**FIGURE 3:**
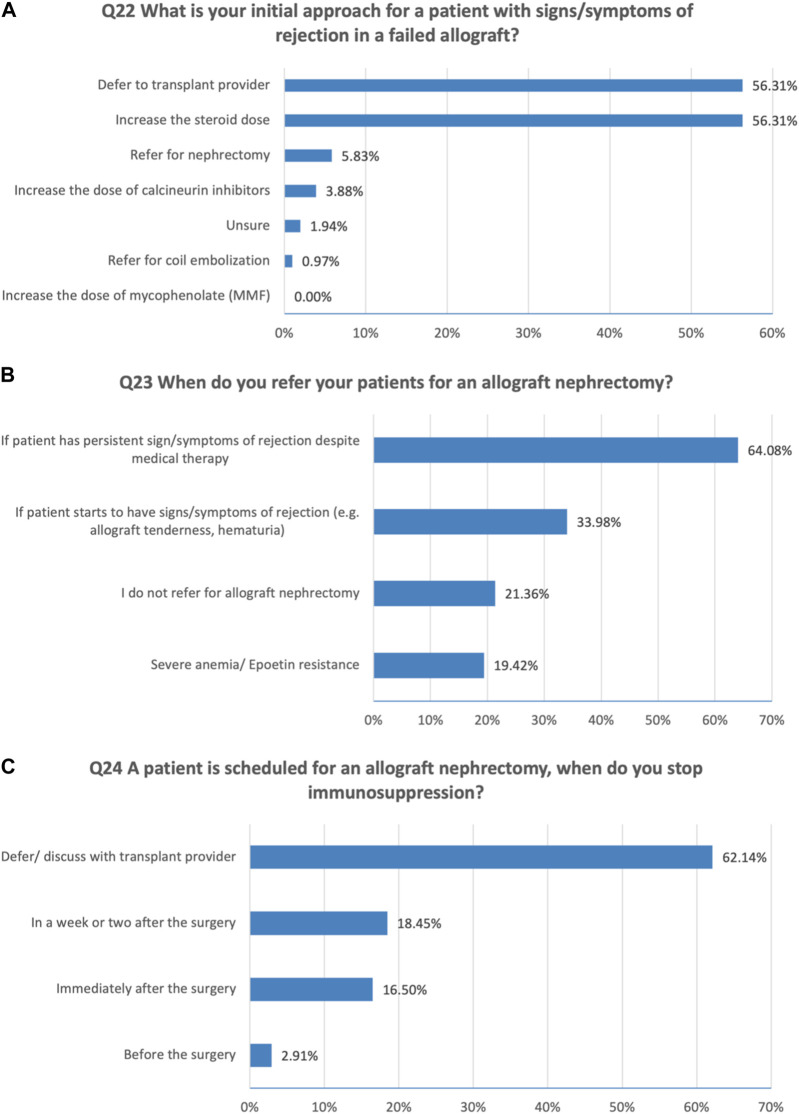
Medical and surgical management of failing kidney allograft. **(A)** Initial approaches to a patient with signs/symptoms of rejection in a failed allograft. **(B)** Timing of referral for an allograft nephrectomy. **(C)** Immunosuppression cessation approaches when a patient is scheduled for an allograft nephrectomy.

Regarding the initial approaches to a patient with signs/symptoms of rejection in a failed allograft (multiple choice question), the majority would increase the steroid dose (56%) and defer to the transplant provider (56%) (Figure 3A).

To be more specific, we asked about the timing of referral for an allograft nephrectomy (multiple choice question). 64% referred patients for nephrectomy if there are persistent signs or symptoms of rejection (e.g., allograft tenderness, hematuria) despite medical therapy (Figure 3B).

For adjusting immunosuppression when a patient is scheduled for an allograft nephrectomy, 62% defer and discuss with transplant provider, 18% reported a week or two after the surgery, 17% reported immediately after the surgery (Figure 3C).

### Differences Across Affiliation Type and Geographical Area

Compared to respondents with a private affiliation, respondents who had an academic affiliation reported a higher percentage of dialysis patients with failed kidney allografts in their practice. Respondents with an academic affiliation were more likely to communicate with transplant centers regarding immunosuppression cessation and less likely to monitor urine output (Table 1). For the initial approach for a patient with signs/symptoms of rejection in a failed allograft, respondents with an academic affiliation were more likely to defer to the transplant provider.

**TABLE 1:** Responses by affiliate and location.

		Affiliate			Location		
Questions	Responses	Private (*n* = 61) (%)	Academic (*n* = 42) (%)		Rural (*n* = 18) (%)	Urban (*n* = 79) (%)	
What percentage of dialysis patients in your practice have failed kidney allografts?	1%–5%	65.6	40.5	*p* = 0.016	72.2	51.9	*p* = 0.581
	11%–20%	1.6	16.7		5.6	7.6	
	6%–10%	29.5	38.1		22.2	35.4	
	>20%	1.6	0.0		0.0	1.3	
	None	1.6	4.8		0.0	3.8	
How often do you or your team discuss transition of care with the transplant team for patients with a failed allograft?	Never	9.8	4.8	*p* = 0.794	5.6	8.9	*p* = 0.053
	Rarely	19.7	23.8		44.4	15.2	
	Sometimes	44.3	45.2		33.3	48.1	
	Always	26.2	26.2		16.7	27.8	
How comfortable do you feel managing immunosuppression with a failing allograft (patient not on dialysis)?	Very comfortable	23.0	21.4	*p* = 0.236	11.1	22.8	*p* = 0.226
	Comfortable unless complications develop	63.9	52.4		77.8	55.7	
	Not comfortable and need guidance	13.1	26.2		11.1	21.5	
What is your initial approach for reduction of immunosuppression in a patient with a failing allograft?	Stop antimetabolites first	67.2	50.0	*p* = 0.108	61.1	58.2	*p* = 0.427
	Stop calcineurin inhibitors first	9.8	4.8		16.7	6.3	
	Stop prednisone first	4.9	7.1		5.6	6.3	
	Defer to transplant nephrologist	18.0	38.1		16.7	29.1	
Do you communicate with transplant centers regarding Immunosuppression cessation?	Yes, always	49.2	73.8	*p* = 0.015	27.8	65.8	*p* = 0.004
	Sometimes	32.8	23.8		61.1	22.8	
	No, I make the decision myself	18.0	2.4		11.1	11.4	
If you manage immunosuppression, do you monitor calcineurin inhibitor levels?	Yes	73.8	54.8	*p* = 0.066	88.9	60.8	*p* = 0.063
	No	21.3	28.6		11.1	26.6	
	Not applicable	4.9	16.7		0.0	12.7	
If you manage immunosuppression, do you monitor urine output?	Yes	70.5	57.1	*p* = 0.029	61.1	64.6	*p* = 0.151
	No	26.2	23.8		38.9	22.8	
	Not applicable	3.3	19.0		0.0	12.7	

Compared to respondents living in rural areas, respondents living in urban areas were more likely to communicate with transplant centers regarding immunosuppression cessation. No differences were found for responses to other questions across affiliation type and geographical area.

## Discussion

This contemporary survey of primarily U.S. adult general nephrologists highlights challenges and practice patterns in the care of patients with failing allografts. Our data reflect the perspectives of general nephrologists who practice in private and academic settings and demonstrate a wide range of practices in the management of a failing allograft. Such heterogeneity speaks to the need for clear and accessible recommendations to guide collaborative co-management of this important and vulnerable patient group.

While listing patients with failing allografts for a subsequent transplant was the primary concern of the majority of nephrologists in this survey, more than a third were cognizant of the increased medical complexity, mortality, and complex psychosocial issues amongst patients with a failing allograft, which are well established in literature [4–6, 11]. It is clear that this patient population needs special attention which may present an additional burden and challenge for busy practices.

Our study highlights that only 39% of respondents noted that patients were always referred back to general nephrologists before being started on dialysis after allograft failure. Establishing care with a general nephrologist before dialysis could improve the patient’s experience and transition in a psychologically challenging time. Furthermore, well-planned transitions of care can help in early dialysis access creation, which in turn can reduce morbidity and mortality [12]. This transition should ideally not be an abrupt change in care teams but is perhaps best suited for a period of co-management between the transplant team and general nephrology, with visits alternating between both practices. This transition needs to incorporate a multidisciplinary team approach and includes nurses, dieticians, pharmacists, and social workers. Educational programs of dialysis modalities need to be part of the clinic. Champion access and the involvement of surgeons and radiologists are important to increase the rates of fistulas. A key strategy for this transition is to start early in the post-transplant course, so general nephrologists maintain their relationships with their patients.

Notably, only 24% of respondents reported that more than half of their patients with a failing allograft were referred for re-transplantation. The majority (77%) of nephrologists felt that patients with a failing allograft were more likely to be referred for transplant as compared to patients with native kidney CKD. Transplant centers have a mutual interest in referring appropriate candidates back for re-transplantation. Based on that, transplant centers do frequently relist patients before going back to dialysis. This is consistent with the finding that there were higher rates of referral for relisting for failed allografts than native CKD patients. It is also possible that patients with failed allografts would try to avoid going back to dialysis and could themselves be more active in trying to get relisted for re-transplantation. Notably, a recent study utilizing the Austrian Dialysis and Transplant Registry showed that the survival benefit of a second kidney transplant is conditional on the wait time since the loss of the first graft, highlighting the necessity of early referral [13].

In terms of communication regarding the transition of care for patients with failed allografts, our data demonstrated that 29% of the general nephrologists rarely or never discussed the care with transplant providers. Urban and academic nephrologists reported significantly better communication with transplant centers regarding immunosuppressant cessation than rural and private nephrologists.

Similar to transplant providers surveyed in an earlier KRAFT survey, general nephrologists felt that minimizing the risk of sensitization for a subsequent transplant is the most important reason to continue immunosuppression medications [9]. The strategy of slowly tapering off immunosuppression has been shown to reduce sensitization following graft failure [14, 15]. Similar to transplant providers, general nephrologists endorsed a strong preference towards first stopping antimetabolites when reducing immunosuppression.

Our study has several limitations. First, our participants represent a subset of the nephrology community and their responses may not be generalizable to the other clinicians. Second, we were not able to calculate the percentage of responses to the survey due to the fact that the survey was posted online through the HUB.

In summary, this survey of general nephrology clinicians highlights a wide range of concerns in the care of patients with failing allografts. Clear areas of opportunity exist for better communication with transplant centers, early referral for a subsequent transplant, and early involvement of general nephrology providers in the care of patients with failing allografts. This study highlights the need to establish and disseminate best practice recommendations, along with structured programs for early involvement of nephrologists in the care of patients with failing allografts and for building a strong network of communication with transplant centers.

## Data Availability

The original contributions presented in the study are included in the article/supplementary material, further inquiries can be directed to the corresponding author.

## Abbreviations

AST, American Society of Transplantation; KPCOP, Kidney Pancreas Community of Practice; CKD, chronic kidney disease; CNIs, calcineurin inhibitors; KRAFT, Kidney Recipients with Allograft Failure - Transition of Care; MMF, mycophenolate mofetil.
